# Ovarian Epithelial-Stromal Interactions: Role of Interleukins 1 and 6

**DOI:** 10.1155/2011/358493

**Published:** 2011-06-26

**Authors:** Kamisha T. Woolery, Patricia A. Kruk

**Affiliations:** ^1^Department of Pathology & Cell Biology, MDC 11, University of South Florida, 12901 Bruce B. Downs Blvd., Tampa, FL 33612, USA; ^2^H. Lee Moffitt Cancer Center, Tampa, FL 33612, USA

## Abstract

Ovarian epithelial cancer is the most lethal gynecologic malignancy. The high mortality is attributed to the fact that most cases typically present in late stage when ovarian cancer (OC) has already spread beyond the ovary. Ovarian epithelial cancer cells are shed into intraperitoneal ascites and easily disseminate throughout the peritoneal cavity with preferential metastasis to the omentum, peritoneum, and local organs. Understanding how ovarian epithelial cells interact with and modulate their microenvironment can provide insight into the molecular mechanism(s) involved with malignant transformation and progression which may eventually identify novel diagnostic, prognostic, and therapeutic targets. The objective of this paper is to provide a brief consideration of ovarian surface epithelial-stromal interactions in regard to normal physiological function and tumor progression as influenced by two potentially key interleukins, interleukins-1 (IL-1) and -6 (IL-6), present in the microenvironment. Lastly, we will consider the clinical implications of IL-1 and IL-6 for OC patients.

## 1. Introduction

Ovarian cancer (OC) is the leading cause of gynecologic cancer death in women in the US. It is the fourth leading cause of cancer death among women after lung, breast, and colorectal cancer and is associated with a 1.7% lifetime risk [1–4]. It is estimated that 23,100 new cases are diagnosed in the US annually [4] and that 14,000 women die annually from this disease. The one-year survival rate for OC can be as high as 79% and when diagnosed in an early stage the 5-year survival rate is almost 95% [4]. However, when diagnosed at a later stage, the 5-year survival is generally no better than 35% [4]. Optimal cytoreduction followed by platinum-based chemotherapy remains the mainstay of therapy in the management of advanced epithelial ovarian cancers [2]. However, while the response rate to primary chemotherapy can be as high as 76%, response rate is dramatically reduced after relapse of disease [5]. Platinum resistance, defined as disease recurrence less than six months from completion of therapy, is an important prognostic predictor. Patients with platinum-resistant tumors have a response rate of less than 10% when retreated with platinum compounds [6, 7] and alternative options also have poor response rates of 18–30% [8–15].

Ovarian surface epithelium-(OSE-) derived tumors account for 90% of malignant ovarian tumors with serous tumors accounting for over 30% of all OSE-derived cancers [16]. The majority of ovarian cancers are sporadic in origin, but about 10% of all epithelial ovarian carcinomas are associated with a hereditary predisposition and are characterized by an increased incidence and earlier onset of disease [17]. Epidemiological studies suggest that besides race [18] and familial history of breast or ovarian cancer [19–21], events associated with OSE traumatization may result in aberrant OSE growth leading to ovarian epithelial carcinogenesis [22]. Thus, increased age, reproductive history (nulliparity), early menarche, late menopause, and fertility drug use increase the risk for OC. In contrast, suppression of ovulation by pregnancy, lactation, or oral contraceptive use decrease the risk for OC. Lifestyle factors including dietary fat intake and smoking may also increase the risk for OC while dietary intake of vitamins A, C, D, and E may protect against OC [23–25]. 

The high mortality associated with this disease is generally attributed to the fact that most cases typically present in late stage when OC has already spread beyond the ovary, that the disease is generally asymptomatic in early stages, and that an effective screening test is lacking [25, 26]. In addition to involving the ovary, ovarian epithelial cancer cells are shed into intraperitoneal ascites fluid and easily disseminate throughout the peritoneal cavity with preferential mestastasis to the omentum and peritoneum as well as other local organs. 

The etiology of OC is not completely known and can be attributed to genetic instability associated with repeated repair of ovulatory defects, elevated gonadotropin levels with inclusion cysts, or explantation of Fallopian tube material. Understanding how ovarian epithelial cells interact with and modulate their microenvironment contributing to cancer initiation and progression can provide insight into molecular mechanism(s) involved with malignant transformation which may eventually identify novel diagnostic, prognostic, and therapeutic targets. Interleukin-1 (IL-1) and interleukin-6 (IL-6) may prove to be such targets. Both cytokines contribute to the progression of OC by promoting angiogenesis, metastasis, and chemoresistance. The main focus of this paper, then, will be to briefly describe the role of IL-6 in OC progression and to more thoroughly examine the role of the lesser understood IL-1 in relation to OC progression and its capacity as a possible therapeutic target. This paper was conducted using a broad literature survey encompassing the past 25 years of basic and clinical research publications exploring IL-1 and IL-6 as each pertains to ovarian cancer prognosis, progression, and treatment. 

## 2. Ovarian Surface Epithelium: Extracellular Matrix Interactions

Despite the fact that over 85% of ovarian cancers arise from and/or involve the OSE [2, 50], the reasons for the propensity of OC development are still poorly understood. However, due to the common embryonic origin of OSE with the epithelia lining Fallopian tube, endometrium, and cervix, histologic subtypes of OSE-derived tumors include serous, endometrioid, and mucinous tumors, respectively. Likewise, the expression of stromal characteristics by OSE cells may be related to their shared mesodermal origin from the coelomic epithelium and to their close developmental relationship to ovarian stromal fibroblasts. For example, OSE express both vimentin and keratin intermediate filaments characteristic of connective tissue and epithelium, respectively [51]. Further, when maintained on collagen type I gel, OSE cells undergo a modulation into a spindle-like morphology which accompanies collagen gel contraction [52]. In contrast, OSE cells invade Matrigel and can remodel extracellular matrix (ECM) by secretion of collagenase, gelatinase and stromelysin as well as produce laminin and collagens I, III and IV [53, 54]. Consequently, the interaction with stroma and the ability of OSE to produce, lyse, and reconstruct ECM indicates that OSE has the capacity to undergo epithelial-mesenchymal conversions which may not only be necessary for restoring the integrity of the OSE following ovulatory damage, but may also contribute to invasion and metastasis with malignant transformation of the OSE. 

## 3. Ovarian Cancer and Epithelial-Mesenchymal Transition

Epithelial-mesenchymal transition (EMT) is characterized by the disruption of cellular junctions and loss of cellular polarity resulting in morphologic modulation into a fibroblastic phenotype accompanied by increased cell motility and invasion [55]. Embryonically, EMT contributes to gastrulation, and in the ovary to gonadal development [56]. During tissue repair following ovulation, OSE undergoes EMT in response to its local microenvironment into a fibroblast-like phenotype which promotes OSE migration, proliferation, and matrix remodeling to repair the ovulatory defect. “Incessant ovulation” proposes that repeated injury to the OSE with subsequent rounds of cellular proliferation cannot only lead to the acquisition of replicative DNA errors contributing to genetic instability and ensuing malignant transformation [57], but also to chronic inflammation [58]. Such inflammation is associated with oxidative stress and recruitment of activated immune cells, including macrophages, T cells, B cells, natural killer cells, neutrophils, and granulocytes [59] resulting in a locally marked increase in cytokines/chemokines (interleukins and tumor necrosis factors) and matrix-remodeling enzymes (plasminogen activators and collagenases) that can also promote tumor formation [57]. 

EMT and subsequent mesenchymal-epithelial (MET) transitions associated with alterations of E-cadherin expression [60] are among the most dramatic examples of OSE plasticity in response to its microenvironment and represent critical steps in ovarian tumor progression. Owing to the mesodermal origin of OSE, normal OSE express N-cadherin, but not E-cadherin. However, in contrast to loss of E-cadherin expression with tumor progression in most epithelial cancers, E-cadherin is often reexpressed in OSE lining crypts and inclusion cysts as well as in benign, borderline, and primary ovarian cancers [60]. Importantly, given that OC metastasizes by shedding into the peritoneal cavity as single cells or cell clusters, transient E-cadherin re-expression maintains OC aggregate formation and survival in the peritoneal cavity [61]. OC aggregates, mesothelial cells, and surrounding blood cells all secrete cytokines that support OC survival in ascites so that both autocrine and paracrine mechanisms sustain OC EMT in ascites fluid and promote establishment of metastatic disease. The reverse process of MET results from loss of transient E-cadherin expression so that established metastasticovarian cancers are frequently devoid of E-cadherin [62]. Pathways promoting EMT in OSE rely on complex interactions between OSE and its extracellular components and consist of autocrine and paracrine interactions with hormones and cytokines including transforming growth factor-*β* [63], epidermal growth factor [64], hepatocyte growth factor [65], endothelin-1 [66], and bone morphogenic protein 4 (BMP4) [67]. Interestingly, epidermal growth factor/epidermal growth factor receptor signaling induces EMT in cultures of OC cell lines and is also associated with enhanced expression and secretion of IL-6, resulting in increased cellular motility [68].

In addition to promoting a migratory and invasive phenotype, EMT may contribute to patient mortality by conferring paclitaxel resistance in epithelial ovarian cancer cells [69], inducing myocyte differentiation into CD14+/KDR+ proangiogenic cells thereby promoting tumor angiogenesis and vascularization [70], and inducing the differentiation of stromal mesenchymal stem cells into tumor associated fibroblasts that support disease progression through matrix remodeling [41]. In this last context, it is interesting that both *in vivo* and *in vitro* studies indicate that IL-6 produced by stromal tumor associated fibroblasts also enhances OC cell proliferation through activated signal transducer and activator of transcription 3 (STAT3) signaling [41].

While nonsteroidal anti-inflammatory drugs (NSAIDS) have failed to protect against development of OC [71, 72], inflammation may still contribute to the clinical development of OC. In addition to presenting as an inflammatory disease, inflammation of the ovarian epithelium has been associated with increased risk for OC [58, 73–75] and endometriosis is associated risk for clear cell carcinoma of the ovary [71, 76–79]. Consequently, the potential clinical contribution of inflammatory mediators, IL-6 and IL-1, for ovarian tumor initiation and progression should not be overlooked.

## 4. Interleukin-6

IL-6 is a pleiotropic cytokine that plays a major role in the immune system in response to injury and infection, as well as, inflammation [45]. IL-6 is produced by T cells, monocytes, fibroblasts, endothelial cells, and keratinocytes [80]. Depending on the experimental system, IL-6 has been suggested to have both proinflammatory and anti-inflammatory properties *in vitro* and *in vivo *[81]. However, it appears that IL-6 acts predominately as an anti-inflammatory and immunosuppressive cytokine by directly suppressing IL-1 and tumor necrosis factor-*α* (TNF-*α*), inducing release of glucocorticoids, and inducing natural antagonists to IL-1 and TNF-*α* [81]. In an *in vitro *prostate cancer model, cancer-associated fibroblasts secreted higher amounts of IL-6 than normal fibroblasts which resulted in an increased proliferation of normal epithelial cells and increased endothelial cell migration towards the cancer-associated fibroblasts and/or their conditioned media [82]. It is not surprising then that Spaeth et al. [41] found that tumor-associated fibroblasts derived and differentiated from mesenchymal stromal stem cells produced and secreted IL-6 that similarly promoted OC cell proliferation [41].

In the normal ovary, aside from production by activated stromal immune cells, IL-6 is produced by granulosa cells [83] and OSE cells [84, 85]. However, neoplastic ovarian cells also routinely overexpress IL-6 *in vitro *[84–86] and greater amounts of IL-6 are present in the cystic fluid of malignant tumors when compared to cystic fluid of benign tumors [80]. Likewise, OC ascites are rich in IL-6 [46, 87–89]. Consequently, elevated levels of IL-6 in the blood and ascites are associated with poor prognosis in OC [90]. Interestingly, elevated serum IL-6 levels are uniquely associated with OC compared to other gynecological malignancies and although this is a much less sensitive biomarker for OC than CA125 [46], it may still serve as a useful prognostic indicator of disease. 

While the role of IL-6 in the etiology of OC is not fully understood, we might predict that IL-6  contributes to OC by promoting angiogenesis, tumor invasion, and chemoresistance. Specifically, *in vivo* treatment with IL-6-induced angiogenesis and enhanced endothelial cell-mediated migration of human ovarian carcinoma cells [42]. IL-6 may also be involved in the tumorigenic processes of OC cell lines by increasing their capacity to secrete matrix metalloproteinase-9 (MMP-9) [43]. Further, OC cell lines cultured with IL-6 demonstrate increased chemotactic and/or chemokinetic activity and increased overall invasiveness [44]. Overexpression of IL-6 is also associated with chemoresistance in OC cells [46, 47]. *In vitro* studies of OC cell lines further show that autocrine production of IL-6 decreased their responsiveness to cisplatin and paclitaxel [45].

Interestingly both IL-6 and IL-1 levels are elevated in ascites. *In vitro* studies show that treatment of granulosa cells with IL-1 increases IL-6 production in a dose-dependent manner [83] and, likewise, treatment of OC cell lines with IL-1*β*, lead to increased secretion of IL-6 [91]. Consequently, the contribution of IL-1 to initiate and promote OC progression by regulating IL-6 expression deserves consideration.

## 5. Interleukin-1

There are 11 members of the IL-1 cytokine family that have proinflammatory or anti-inflammatory activity. The most thoroughly studied cytokines from this family are two agonist cytokines: IL-1*α* and IL-1*β* and one antagonist cytokine: interleukin-1 receptor antagonist (IL-1Ra). Though all of these cytokines are associated with chronic inflammatory diseases (CIDs), IL-1*β* appears to be the primary mediator of inflammation in CIDs [39].

IL-1*β* is synthesized in a precursor form as a 31 kD protein that is cleaved by caspase-1 into its active 17 kD mature secreted form [92]. IL-1*β* is mainly produced by monocytes and macrophages, but can also be produced by endothelial cells, fibroblasts, and epidermal cells in response to bacterial or innate immunity stimulation [93]. Both normal and malignant epithelial ovarian cells also produce IL-1 [90], although activated immune cells in the stroma remain the major source of IL-1 [94]. Constitutive production of IL-1*β* by ovarian carcinoma cells [95] enhances their invasion capacities by increasing expression of MMP-1 [36] and stimulating production of proangiogenic factors such as vascular endothelial growth factor (VEGF) [37]. Additionally, fibroblasts cultured with conditioned media from a highly metastatic OC cell line or with IL-1*β* induced transformation of the fibroblasts to myofibroblasts expressing *α*-smooth muscle actin (*α*-SMA) [38]. This coincided with induction of myofibroblast expression of fibroblast activation protein (FAP), a cell-surface serine protease with capacity to remodel ECM by cleaving collagen type 1 [38], thereby promoting OC cell migration and invasion. 

IL-1*α* is likewise synthesized in a precursor form as a 31 kD protein that is cleaved by Ca^2+^-dependent protease calpain into an active 17 kD mature cell-associated form [28, 92]. IL-1*α* is produced by monocytes, macrophages, epithelial cells, keratinocytes, and fibroblasts [96]. However, because it remains mainly cell-associated, IL-1*α* induces less inflammation and angiogenesis than IL-1*β* [27]. Further, since IL-1*α* is generally secreted to a lesser extent than IL-1*β*, it is not commonly detected in bodily fluids except when released from necrotizing cells in cases of severe inflammation [28]. In its cell-associated form, IL-1*α* mainly acts as a tumor suppressor in malignant cells by recruiting immunocompetent cells to the tumor microenvironment and assisting in an immunologic response to combat tumor growth [28–31]. IL-1*α* tumor suppressor characteristics have been further confirmed *in vivo *where IL-1*α* produced by tumorigenic fibroblasts decreased the number of tumor growths, as well as increased tumor rejection by facilitating activation and expansion of helper T cells [32, 33]. Consequently, in OC, IL-1*β* generally promotes invasiveness, tumor angiogenesis and induces immune suppression while IL-1*α* reduces tumorigenicity by inducing antitumor immunity [97]. 

Lastly, IL-1Ra is an antagonist that inhibits IL-1*α* and IL-1*β* from binding to both IL-1 receptors, but does not activate IL-1 receptor cell signaling cascades. IL-1Ra is secreted by the same cell types as IL-1*α* and IL-1*β* including monocytes, macrophages, and OSE [98]. Further, high levels of IL-1Ra in cultured tumor-derived macrophages compared to cultured tumor-derived ovarian epithelial cells [99] suggest that the major contributors of IL-1Ra are activated macrophages. IL-1Ra is excessively abundant in the ascites of late-stage OC patients compared to IL-1Ra levels in ascites and/or serum levels from early-stage OC and benign ovarian tumors [40]. Higher levels of IL-1Ra in ascites and/or serum appear associated with poor prognosis and reduced overall survival [40]. Since IL-1Ra does not activate IL-1 receptor cell signaling cascades and IL-1Ra production can be induced by IL-1*β* and/or IL-1*α*, it is plausible that the increased IL-1Ra in the ascites and the associated poor prognosis is due to secreted IL-1*β* signaling [31]. In agreement, Mustea et al. found that increased levels of IL-1*β* in the serum and/or ascites were associated with decreased survival [40]. Therefore, it is imperative to further understand the clinical significance of the IL-1 family in the progression of OC as well as potential treatment targets.

## 6. Clinical Relevance

Phase I and II clinical trials utilizing recombinant human IL-6 in conjunction with chemotherapy increased OC patient platelet count alleviating thrombocytopenia [48, 49], thereby comprising adjuvant therapeutic benefits of IL-6. As stated earlier, IL-1 increases production of IL-6 [83, 91]; therefore, due to this interrelationship, studies investigating the effect of IL-1 on the development of OC are clinically important. A better understanding of the IL-1 cytokines' functions in relationship to OC may help to identify novel prophylactic drug treatments and/or treatments after disease occurrence. 

As described earlier, IL-1*α* has tumor suppressor functions that should be further investigated in OC; however, the effectiveness of IL-1*α* as a treatment option is likely limited to early onset of disease. When released in its precursor form, such as from necrotic tumor cells under hypoxic conditions commonly seen in OC [96], IL-1*α* can promote an inflammatory response perpetuating the chronic inflammation associated with the onset of disease [96]. Yet, when released in its cleaved form, IL-1*α* lacks the ability to interact with its receptor [96] so that treatment with the precursor form would be expected to elicit a proinflammatory response and treatment with the cleaved form would elicit no response.

On the other hand, *in vitro* studies have shown that IL-1*α* increases 11*β*-hydroxysteroid dehydrogenase type 1 (11*β*HSD1) mRNA expression in human primary OSE [100] leading to increased rates of cortisone to cortisol conversion by OSE [101] promoting cortisol's anti-inflammatory potential [102]. However, since 11*β*-hydroxysteroid dehydrogenase type 2 (11*β*HSD2) activity is greater in OC tissue compared to normal ovarian tissue [103], it is not surprising that OC cell lines treated with IL-1*α* *in vitro* induced 11*β*HSD2 gene expression leading to the reverse conversion of cortisol to cortisone inactivating the anti-inflammatory actions of 11*β*HSD1 and, thereby, supporting tumor cell proliferation [104]. Consequently, the IL-1*α*-induced switch from 11*β*HSD1 to 11*β*HSD2 associated with malignant transformation confers a growth advantage to OC cells [105] and limits the therapeutic potential of IL-1*α*.

A phase I clinical trial showed recombinant human IL-1*α* treatment having minor antitumor effect in advanced recurrent ovarian cancer [106]. Nonetheless, clinical trials showed that recombinant human IL-1*α* treatment accelerated platelet recovery during chemotherapy and reduced carboplatin-induced thrombocytopenia in patients with recurrent OC [34], as well as in patients with ovarian and other cancer types (gastrointestinal, breast, melanoma, lung, head and neck, sarcoma, and prostate) who did not receive concomitant chemotherapy [35]. However, IL-6 is an effective hematopoietic growth factor and plays a direct role in megakaryocyte differentiation into platelets [48]. Therefore, it can be hypothesized that the platelet increase seen in response to recombinant human IL-1*α* treatment could also be due to IL-1-induced production of IL-6 [35]. Therefore, more clinical trials should be completed to test the efficiency of IL-1 and/or IL-6 to ameliorate thrombocytopenia.

In contrast, due to IL-1*β*'s cancer-promoting activities, inhibiting IL-1*β* function represents a promising new avenue for therapeutic intervention. Developing novel IL-1*β* antagonists that block IL-1*β*'s ability to bind to its receptor should suppress IL-1*β*'s VEGF-mediated angiogenesis, invasiveness, and chemoresistance. While published clinical trials of IL-1*β* as a therapeutic target in treatment of cancer is limited, a phase I trial showed treatment with recombinant human IL-1*β* elevated platelet levels in patients with gastrointestinal cancer [107]. However, as stated earlier, IL-1's ability to increase platelet levels may be partially due to inducing production of IL-6. However, due to the aggressiveness of OC and IL-1*β*'s promotion of cancer progression, it may prove more beneficial to evaluate IL-1*β* as a treatment target rather than as a therapeutic agent. Therefore, as a treatment target effective agents would likely mimic the action of IL-1*β*'s natural antagonist and/or neutralize IL-1*β*. Currently, a recombinant IL-1Ra, *Anakinra*, which blocks IL-1*β* and IL-1*α* from binding to their receptors, is used for the treatment of rheumatoid arthritis [108]. Likewise, the fusion glycoprotein, *Rilonacept*, which works by binding to IL-1*α* and IL-1*β* with high affinity to neutralize both molecules is yet another anti-inflammatory therapy currently in use [108]. Lastly, *Canakinumab*, a fully humanized monoclonal antibody highly specific for IL-1*β*, is currently in clinical use to suppress inflammation [108]. Consequently, these approved or similarly targeted inflammatory medications may be valuable for their capability to reduce IL-1-induced disease processes that promote OC.

## 7. Conclusion

In conclusion, IL-1 and IL-6 significantly impact the ovarian cancer physiologic and structural microenvironment. Specifically, either cytokine can promote proliferative, migratory and invasive changes in ovarian cancer cells. Clinically recombinant IL-1 and IL-6 may alleviate chemotherapy-induced thrombocytopenia. Furthermore, IL-1*β* neutralizing treatments, such as those employed in autoinflammatory diseases, combined with chemotherapy may prove beneficial to reduce IL-1*β*-mediated angiogenesis and metastasis. 

This paper has explored the cancer-promoting aspects of IL-1 and IL-6 summarized in Table 1, as well as, their beneficial characteristics in alleviating a chemotherapy side effect. There is a delicate balance that must be achieved between using IL-1 and IL-6 for therapeutic purposes, while also abrogating the cancer-promoting characteristics of each cytokine. Consequently, further experimental and clinical studies of IL-1 and IL-6 are warranted and may provide a better understanding of interleukin-associated changes to the microenvironment associated with ovarian cancer progression leading to improved prognosis by novel treatment options.

## Figures and Tables

**Table 1:** Proposed functions of IL-1 and IL-6 in OC.

Cytokine	Inflammatory fnction	Proposed function in ovarian cancer	Model	Ref.
IL-1*α*	Proinflammatory	(i) Stimulate angiogenesis	(i)* In vitro/In vivo *	[27]
		(ii) Recruit immunocompetent cells	(ii)* In vitro/In vivo *	[28–33]
		(iii) Increase platelet count	(iii) Phase I clinical trial	[34, 35]

IL-1*β*	Proinflammatory	(i) Increase expression of MMP-1	(i)* In vitro *	[36]
		(ii) Stimulate production of proangiogenic factors	(ii)* In vitro *	[37]
		(iii) Promote OC cell migration and invasion	(iii)* In vitro *	[38]

IL-1Ra	Anti-inflammatory	(i) Antagonist to IL-1*α* and IL-1*β*		[39]
		(ii) Abundant in the OC ascites	*In vivo*	[31, 40]

IL-6	Anti-inflammatory	(i) Promote OC cell proliferation	(i)* In vitro *	[41]
		(ii) Stimulate angiogenesis	(ii)* In vitro/In vivo *	[42]
		(iii) Enhance endothelial cell migration	(iii)* In vitro/In vivo *	[42]
		(iv) Increase OC cell lines capacity to secrete MMP-9	(iv)* In vitro *	[43]
		(v) Increase chemotactic and/or chemokinetic activity	(v)* In vitro *	[44]
		(vi) Increase invasiveness	(vi)* In vitro *	[44]
		(vii) Induce chemoresistance	(vii)* In vitro/In vivo *	[45–47]
		(viii) Increase platelet count	(viii) Phase I and II clinical trial	[48, 49]

## Abbreviations

CIDs: Chronic inflammatory diseases
BMP4: Bone morphogenic protein 4
ECM: Extracellular matrix
11*β*HSD1: 11*β*-hydroxysteroid dehydrogenase type 1
11*β*HSD2: 11*β*-hydroxysteroid dehydrogenase type 2
IL-1: Interleukin-1
IL-1Ra: Interleukin-1 receptor antagonist
IL-6: Interleukin-6
EMT: Epithelial-mesenchymal transition
FAP: Fibroblast activation protein
MET: Mesenchymal-epithelial transition
MMP: Matrix Metalloproteinase
NSAIDS: Nonsteroidal anti-inflammatory drugs
OC: Ovarian cancer
OSE: Ovarian surface epithelium
*α*-SMA: *α*-smooth muscle actin
STAT3: Signal transducer and activator of transcription 3
TNF-*α*: Tumor necrosis factor-*α*
VEGF: Vascular endothelial growth factor.
